# Transthyretin Cardiac Amyloidosis in a Very Elderly Patient With a History of Inferior Myocardial Infarction: A Case Report

**DOI:** 10.7759/cureus.78752

**Published:** 2025-02-08

**Authors:** Satoshi Kurisu, Hitoshi Fujiwara

**Affiliations:** 1 Department of Cardiology, National Hospital Organization Hiroshima-Nishi Medical Center, Otake, JPN

**Keywords:** coronary artery disease, echocardiography, electrocardiogram, non-invasive test, scintigraphy

## Abstract

Transthyretin cardiac amyloidosis (ATTR-CA) involves the buildup of transthyretin protein in the heart muscle in the form of amyloid fibrils, which can affect heart structure and function. Common ECG findings of ATTR-CA include low QRS voltage and a pseudo-myocardial infarction (MI) pattern, defined as pathological Q waves or QS complexes in two consecutive leads without a history of MI or echocardiographic evidence of akinetic areas. Here, we present a case of ATTR-CA in a very elderly patient, in whom pathological Q waves on ECG were true indicators of a prior inferior MI. A 96-year-old woman with a history of inferior MI presented to her primary care clinic with a one-week history of nocturnal dyspnea. She had undergone coronary stent placement in the distal right coronary artery five years earlier for inferior MI. An ECG revealed abnormal Q waves, ST elevation of 0.5 mm, and T wave inversion in limb leads III and aV_F_, with no significant findings suggestive of left ventricular (LV) hypertrophy. Over a two-year period, QRS voltage progressively decreased in all leads, while the ST-T changes remained unchanged. Transthoracic echocardiogram (TTE) showed LV concentric hypertrophy with an increased wall thickness of 14 mm, except in the infero-septal region. In basal and mid-short-axis views, infero-septal wall motion was severely reduced, with notable wall thinning in contrast to the global LV hypertrophy observed elsewhere - findings consistent with prior inferior MI. The patient was ultimately diagnosed with ATTR-CA based on technetium-99m-pyrophosphate scintigraphy and monoclonal protein detection tests. Clinicians should recognize that pathological Q waves in ATTR-CA do not always indicate a pseudo-MI pattern. When both ECG and TTE suggest an MI pattern, further evaluation for coronary artery disease is warranted as part of the ATTR-CA diagnostic workup. In patients with both ATTR-CA and prior MI, a comprehensive clinical approach addressing both conditions is essential for optimizing prognosis.

## Introduction

Transthyretin cardiac amyloidosis (ATTR-CA) is an infiltrative disease characterized by the deposition of transthyretin as amyloid fibrils in the myocardium [1-4]. In recent years, ATTR-CA has gained significant attention as a cause of heart failure, largely due to advancements in noninvasive diagnostic methods and the development of novel treatment options [1-4]. ATTR-CA is classified into two types: hereditary and wild type [1-3]. The hereditary form results from a mutation in the transthyretin gene, while the wild type is associated with age-related transthyretin misfolding, though its exact mechanism remains unclear. Consequently, many patients with ATTR-CA are at risk for age-related comorbidities [1-4], including coronary artery disease (CAD). However, reports on the coexistence of ATTR-CA and CAD, particularly myocardial infarction (MI), remain scarce [5].

A 12-lead ECG and transthoracic echocardiogram (TTE) are essential for diagnosing both conditions. Common ECG findings in ATTR-CA include low QRS voltage and a pseudo-MI pattern - defined as pathological Q waves or QS complexes in two consecutive leads without prior MI or corresponding echocardiographic akinetic areas [6-8]. TTE findings characteristic of ATTR-CA include increased wall thickness, granular sparkling, and apical sparing of longitudinal strain [1-4]. In contrast, prior MI is typically associated with decreased wall thickness and wall motion abnormalities corresponding to the infarct-related artery [9].

Technetium-99m-pyrophosphate (^99m^Tc-PYP) scintigraphy has recently become a cornerstone in ATTR-CA diagnosis [1-4]. When ^99m^Tc-PYP scintigraphy is positive in the absence of monoclonal protein in serum or urine, its specificity and positive predictive value for ATTR-CA reach 100% [10].

Here, we present a case of ATTR-CA in a very elderly patient, where pathological Q waves on ECG were indicative of a prior inferior MI. This case highlights the simultaneous presence of characteristic ECG and TTE findings for both diseases.

## Case presentation

A 96-year-old woman with a history of inferior MI presented to her primary care clinic with a one-week history of nocturnal dyspnea. She had undergone coronary stent placement in the distal right coronary artery five years earlier. Since then, she has been on regular medical follow-ups and was receiving carvedilol (5 mg), rosuvastatin (5 mg), spironolactone (12.5 mg), and azosemide (15 mg). She also had paroxysmal atrial fibrillation and was on edoxaban (30 mg/day). She had no history of carpal tunnel syndrome or lumbar spinal canal stenosis, nor a family history of heart disease.

The patient reported a weight gain of 3 kg along with bilateral lower extremity edema and was referred to our hospital for cardiac evaluation. On physical examination, her pulse rate was 74 bpm, blood pressure was 136/66 mmHg, body weight was 54 kg, and oxygen saturation was 98%. Obvious jugular vein distension was observed in a sitting position, suggesting increased right atrial pressure.

Laboratory studies revealed impaired renal function with an estimated glomerular filtration rate of 37.1 mL/min/1.73 m² (Table 1). Myocardial enzymes, including creatine kinase, were within normal ranges. However, N-terminal pro-brain natriuretic peptide (NT-proBNP) was elevated to 3,196 pg/mL (reference range: <126 pg/mL).

**Table 1:** Laboratory data

Variable	Result	Reference range
White blood cell count (/μL)	6.0 × 10³	3.3-8.6 × 10³
Red blood cell count (/μL)	3.73 × 10⁶	3.86-4.92 × 10⁶
Hemoglobin (g/dL)	11.9	11.6-14.8
Platelet count (/μL)	153 × 10³	158-348 × 10³
Aspartate aminotransferase (U/L)	21	13-30
Alanine aminotransferase (U/L)	13	7-23
Lactate dehydrogenase (U/L)	259	124-222
Creatine kinase (U/L)	67	41-153
Total protein (g/dL)	7.9	6.6-8.1
Albumin (g/dL)	4.5	4.1-5.1
Blood urea nitrogen (mg/dL)	17.5	8-20
Creatinine (mg/dL)	1.04	0.46-0.79
Estimated glomerular filtration rate (mL/min/1.73 m²)	37.1	
C-reactive protein (mg/dL)	0.2	0-0.14
N-terminal pro-brain natriuretic peptide (pg/mL)	3,196	<126
Troponin-I (pg/mL)	66	0-26.2
Free light chain kappa (mg/L)	59.9	3.3-19.4
Free light chain lambda (mg/L)	36.9	5.7-26.3
Kappa-to-lambda ratio	1.62	0.26-1.65
Monoclonal protein	Not detected	

**Figure 1:**
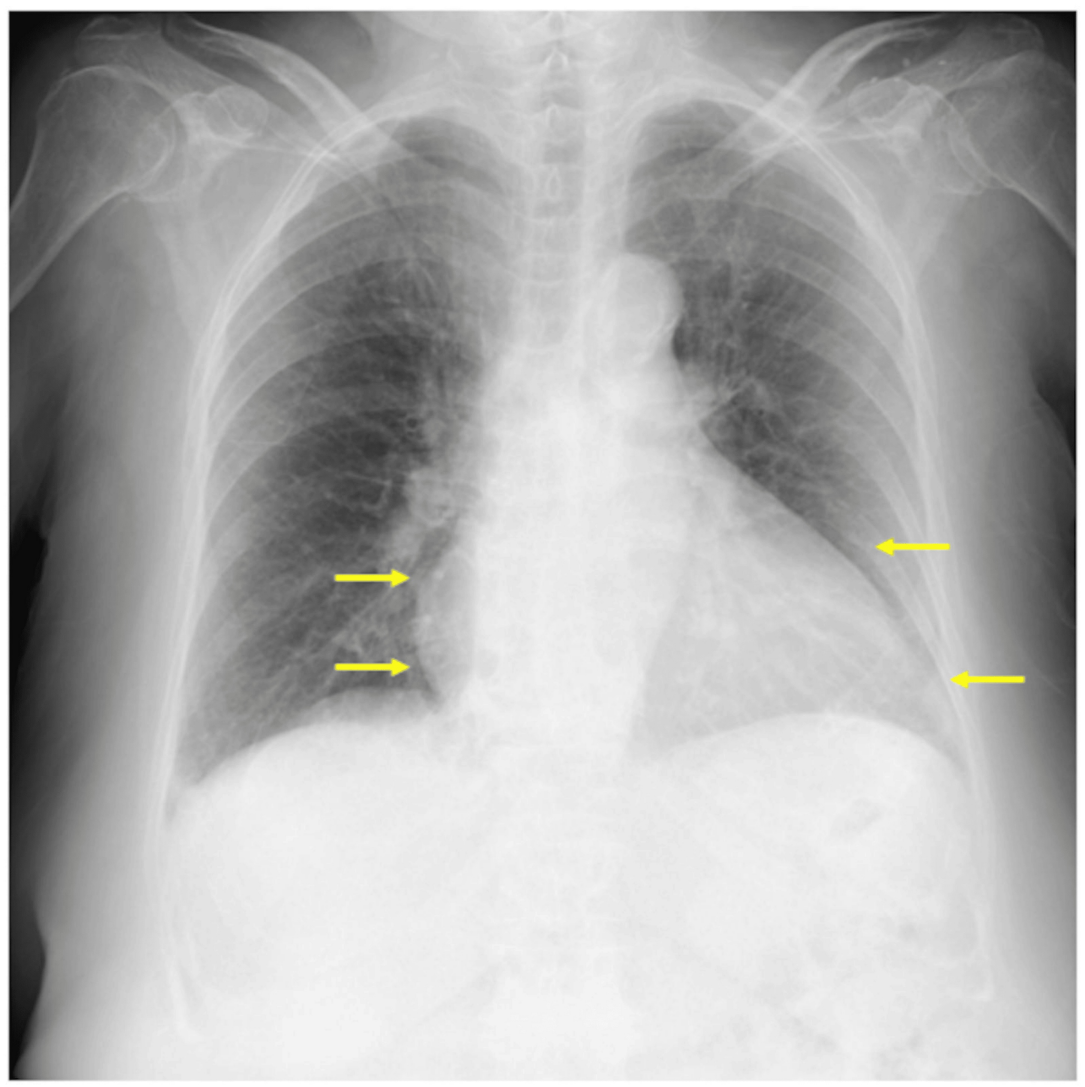
Chest radiograph The image showed mild pulmonary congestion and cardiomegaly (arrows), with a cardiothoracic ratio of 62%.

A chest radiograph showed mild pulmonary congestion and cardiomegaly (Figure 1, arrows), with a cardiothoracic ratio of 62%.

**Figure 2:**
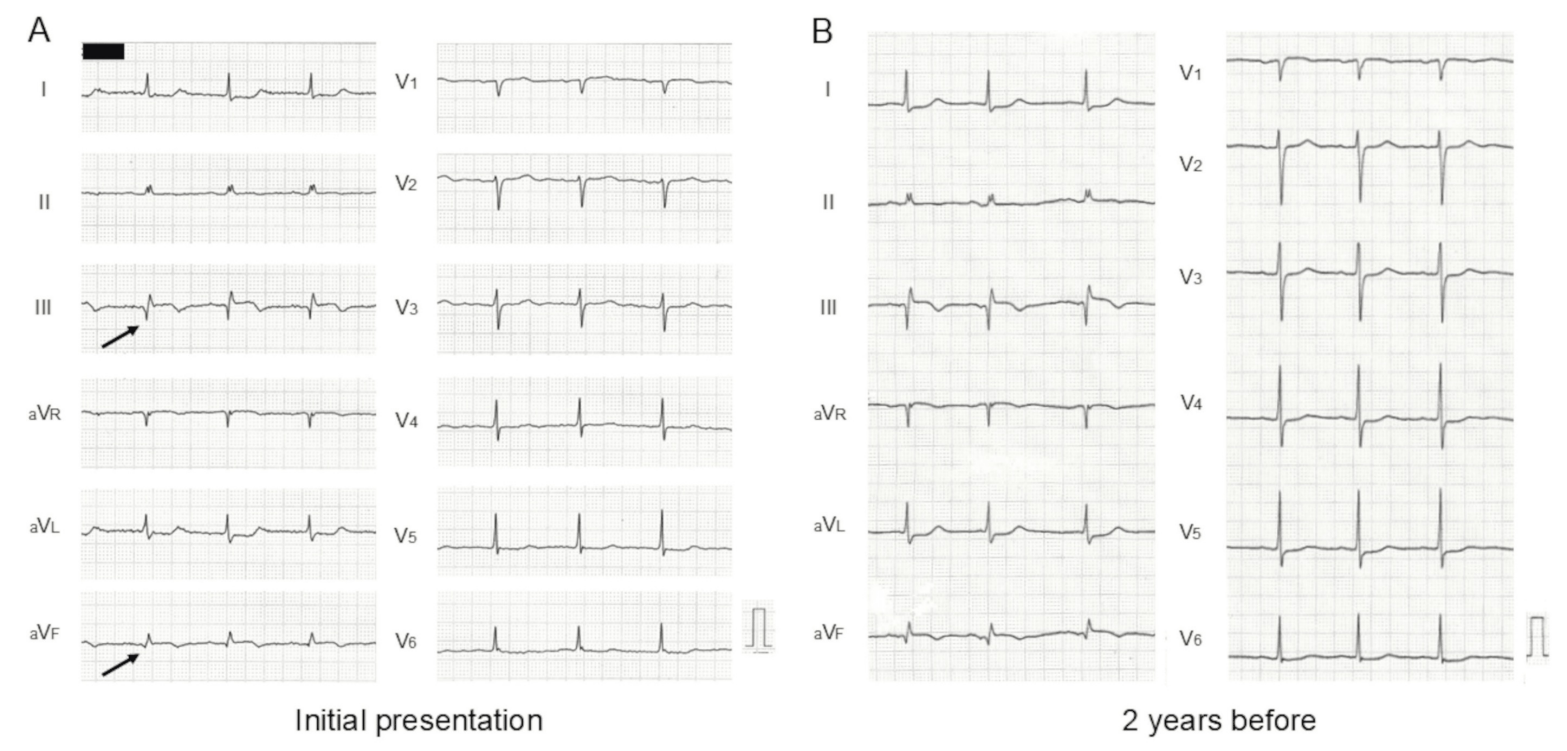
ECGs The ECG showed abnormal Q waves (A, arrows), ST elevation of 0.5 mm, and T-wave inversion in limb leads III and aV_F_, with findings unchanged from two years earlier (B). Over time, QRS voltage decreased across all leads, while ST-T changes remained stable.

An ECG revealed abnormal Q waves (Figure 2A, arrows), ST elevation of 0.5 mm, and T wave inversion in limb leads III and aV_F_. Reciprocal ST depression was observed in limb leads I and aV_L_. No significant ECG findings suggestive of left ventricular (LV) hypertrophy, such as high voltage or a strain pattern, were present. These findings were identical to those seen on an ECG performed two years earlier (Figure 2B). Over this period, QRS voltage decreased in all leads, while the previously noted ST-T changes remained unchanged.

**Figure 3:**
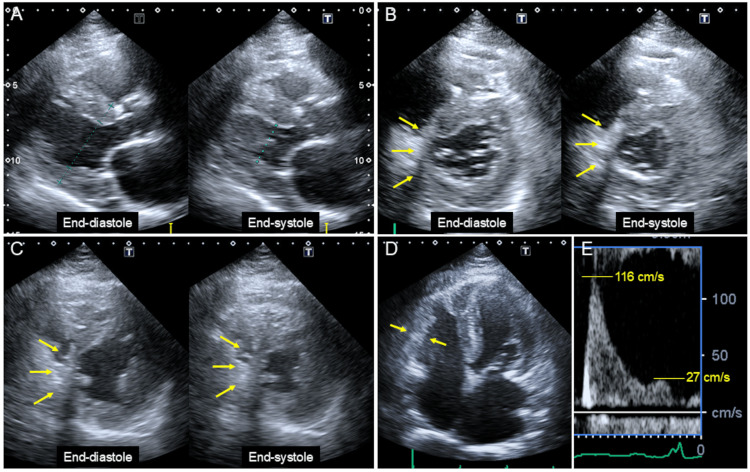
TTE images TTE demonstrated preserved LV systolic function (ejection fraction: 55%) and LV concentric hypertrophy with increased wall thickness of 14 mm (A), except in the infero-septal area. Basal and mid-short-axis views revealed severely reduced infero-septal wall motion and wall thinning, contrasting with global LV hypertrophy (B, C, arrows), consistent with prior inferior MI. The right ventricular free wall was also thickened (D). Doppler studies showed an early-to-late diastolic mitral flow velocity ratio of 4.3 (E), indicating restrictive physiology. MI, myocardial infarction; TTE, transthoracic echocardiogram

A TTE revealed preserved LV systolic function with an ejection fraction of 55% and LV concentric hypertrophy, with an increased wall thickness of 14 mm (Figure 3A), except in the infero-septal area. In the basal and mid-short-axis views, infero-septal wall motion was severely reduced. Infero-septal wall thinning was observed in contrast to the global LV hypertrophy seen in other areas (Figure 3B-3C, arrows), consistent with prior inferior MI. The right ventricular free wall was also thickened (Figure 3D).

Doppler studies showed an early (E) to late (A) diastolic mitral flow velocity ratio of 4.3 (Figure 3E) and an early diastolic mitral annular velocity (e’) of 4.0 cm/s, indicating restrictive physiology (Table 2). The three key parameters recommended in the 2016 diastolic function guidelines [11] were as follows: an average E/e’ of 24.2, a tricuspid regurgitation flow velocity of 3.1 m/s, and a left atrial volume index of 46.4 mL/m². The inferior vena cava diameter at end-expiration was 17 mm, with a collapsibility index of 41%, suggesting volume overload.

**Table 2:** TTE measurements LV, left ventricular; TTE, transthoracic echocardiogram

Variables	Day 1	Day 10
LV end-diastolic dimension (mm)	37	42
LV end-systolic dimension (mm)	27	31
Interventricular septal thickness (mm)	14	14
Posterior wall thickness (mm)	14	14
Left atrial dimension (mm)	43	39
LV ejection fraction (%)	51	53
Early diastolic mitral annular velocity (cm/s)	4	3.8
Early diastolic mitral flow velocity (cm)	116	124
Late diastolic mitral flow velocity (cm)	27	50
Early to late diastolic mitral flow velocity ratio	4.3	2.8
Inferior vena cava diameter (mm)	17/10	10/2
Inferior vena cava collapsibility index (%)	41	80

The combination of low QRS voltage on ECG and LV concentric hypertrophy on TTE raised suspicion of cardiac amyloidosis. The patient was admitted for further cardiac evaluation and treatment.

The patient was treated with an increased dose of azosemide (45 mg/day) immediately after admission. Jugular vein distension and edema resolved completely within four days. The diameter of the inferior vena cava at end-expiration decreased from 17 mm to 10 mm, indicating appropriate volume reduction (Table 2).

Following volume optimization, the patient underwent diagnostic tests for cardiac amyloidosis. The serum troponin I level was elevated at 66 pg/mL (reference range: 0-26.2 pg/mL) (Table 1). Serum and urine immunoglobulin electrophoresis revealed no abnormal bands, and serum-free light chain assays showed a normal kappa-to-lambda ratio of 1.62 (reference range: 0.26-1.65).

**Figure 4:**
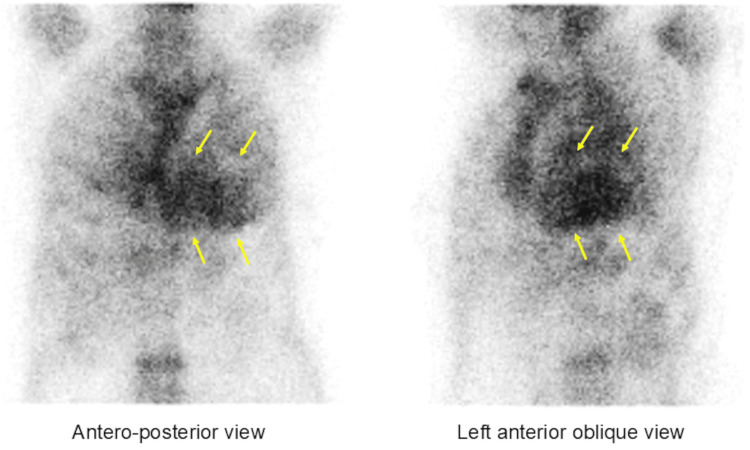
Technetium-99m-pyrophosphate scintigraphic images The scan demonstrated intense myocardial uptake (grade 3), exceeding rib uptake, with a heart-to-contralateral ratio of 1.76.

^99m^Tc-PYP scintigraphy demonstrated myocardial uptake greater than rib uptake (grade 3), with an increased heart-to-contralateral ratio of 1.76 (Figure 4).

At the request of her family, neither an endomyocardial biopsy nor a transthyretin gene sequencing test was performed. Based on these results, the patient was eventually diagnosed with ATTR-CA. She was discharged on day 15 after dose titration of azosemide (30 mg/day). The patient remained in stable condition without recurrence of clinical congestion during the three-month follow-up period.

## Discussion

In this report, we presented a case of ATTR-CA in a very elderly patient, characterized by pathological Q waves on ECG that were truly indicative of prior inferior MI. Notably, characteristic ECG and TTE findings of both diseases were present simultaneously.

Diagnosing ATTR-CA can be challenging due to its varied clinical presentation and confounding comorbidities [1-5], often leading to delayed or missed diagnoses [12,13]. In this case, despite regular medical follow-up, it took five years to diagnose ATTR-CA after inferior MI. The patient eventually developed heart failure, presenting with LV concentric hypertrophy and restrictive physiology.

Wild-type ATTR-CA primarily affects older adults, predominantly men (male-to-female ratio: 25-50:1) [14]. Early symptoms may include carpal tunnel syndrome or lumbar canal stenosis, caused by amyloid fibril deposition [15,16]. However, in this case, the patient was a woman with no history of these conditions, making the diagnosis less obvious during routine medical follow-up. Additionally, she was already 91 years old when she underwent coronary stent placement for inferior MI, which may have contributed to diagnostic inertia over time.

Cardiac biomarkers, including high-sensitivity troponin T, troponin I, and NT-proBNP, are useful in the initial diagnostic evaluation of ATTR-CA [2,4]. However, elevated levels are nonspecific to the disease. Regardless of whether disease-modifying therapies, such as tafamidis, are indicated, early identification using noninvasive tests is crucial for improving patient prognosis.

In recent years, ^99m^Tc-PYP scintigraphy has become a cornerstone of ATTR-CA diagnosis, representing a paradigm shift in diagnostic approaches. Gillmore et al. reported that in patients with grade 2 or 3 myocardial uptake on bone scintigraphy and no monoclonal protein in serum or urine, the specificity and positive predictive value for ATTR-CA were 100% [10]. Castano et al. further demonstrated that a heart-to-contralateral ratio of ≥1.6 was associated with worse survival among ATTR-CA patients [17]. These findings highlight the diagnostic and prognostic significance of ^99m^Tc-PYP scintigraphy for patients with high clinical suspicion of ATTR-CA.

In this case, ^99m^Tc-PYP scintigraphy revealed grade 3 myocardial uptake with a heart-to-contralateral ratio of 1.76, providing strong evidence for a noninvasive diagnosis of ATTR-CA.

Another unique aspect of this case was the coexistence of ATTR-CA and prior MI. Reports on the association between ATTR-CA and CAD remain limited [5]. Hassen et al. recently examined CAD prevalence in 133 ATTR-CA patients, identifying 6 cases of prior ST-elevation MI (STEMI) and 11 cases of prior non-STEMI [5]. However, ECG and TTE images of ATTR-CA complicated by prior MI have rarely been documented.

In this case, we observed a progressive decrease in QRS voltage over a two-year period, while ST-T changes due to prior MI remained unchanged. TTE further revealed infero-septal wall thinning, contrasting with global LV hypertrophy in other regions. These findings suggest that ECG and TTE abnormalities resulting from prior MI can persist despite advanced transthyretin deposition in the myocardium.

The pseudo-MI pattern is a well-known ECG finding in ATTR-CA [6-8]. Murtagh et al. reported pseudo-MI patterns in 47% of patients with biopsy-proven primary cardiac amyloidosis who had no history of MI, with these patterns most commonly appearing in the anterior (36%), inferior (12%), and lateral (14%) leads [18].

In this case, however, the pathological Q waves in the inferior leads were truly indicative of prior inferior MI, rather than a pseudo-MI pattern. This highlights the need for clinicians to differentiate pathological Q waves from pseudo-MI patterns in ATTR-CA. If both ECG and TTE findings suggest MI, further coronary evaluation is warranted as part of the diagnostic workup for ATTR-CA.

In patients with both ATTR-CA and prior MI, a comprehensive clinical management approach addressing both conditions is essential to improve outcomes and prognosis.

## Conclusions

We encountered a case of ATTR-CA in a very elderly patient, presenting with pathological Q waves on ECG that were truly indicative of prior inferior MI. While the pseudo-MI pattern is a common ECG finding in ATTR-CM, clinicians should recognize that pathological Q waves in ATTR-CA do not necessarily indicate a pseudo-MI pattern. If both ECG and TTE findings suggest MI, further CAD evaluation should be performed as part of the diagnostic workup for ATTR-CA. In patients with both ATTR-CA and prior MI, a comprehensive clinical management strategy addressing both conditions is essential to optimize prognosis.
